# Bupropion-Induced Dystonia: A Case Report

**DOI:** 10.7759/cureus.29857

**Published:** 2022-10-03

**Authors:** Tafseer Zahra, Diana Voloshyna, Anan Bseiso, Tanveer Ahamad Shaik, Hayder G Ferman, Meenakshi Sathish, Saman Al Barznji, Faraz Saleem, Muhammad Abu Zar Ghaffari

**Affiliations:** 1 Internal Medicine, California Institute of Behavioral Neurosciences & Psychology, Fairfield, USA; 2 Internal Medicine, University of Michigan, Ann Arbor, USA; 3 Internal Medicine, College of Medicine, Al-Quds University, Jerusalem, PSE; 4 Internal Medicine, College of Medicine, Hebron University, Hebron, PSE; 5 Cardiovascular Medicine, University of Louisville School of Medicine, Louisville, USA; 6 Internal Medicine, College of Medicine, University of Sulaimani, Sulaymaniyah, IRQ; 7 Surgery, Caribbean Medical University School of Medicine, Chicago, USA; 8 Internal Medicine, University of Sulaymaniyah, Sulaymaniyah, IRQ; 9 Internal Medicine, Akhtar Saeed Medical and Dental College, Lahore, PAK

**Keywords:** drug induced dystonia (did), drug induced dystonia, dystonia, antidepressant drug, bupropion

## Abstract

Bupropion is one of the most commonly prescribed antidepressant medications by physicians all over the world. Because of its favorable sexual profile, it is used as an alternative to serotonin reuptake inhibitors (SSRIs). Its significance in smoking cessation is also well recognized. However, it is associated with a few side effects, such as dizziness, anxiety, tremors, nausea, and insomnia. We present the case of a 54-year-old chronic smoker who developed acute facial dystonia involving the temporomandibular joint (TMJ) after being prescribed 300 mg of bupropion. The Naranjo scale was used to assess the probability of bupropion-induced dystonia. Following the diagnosis, the drug was stopped, and the dystonia completely resolved within one week. At her follow-ups, the patient was found to have no recurrence of dystonia.

## Introduction

A norepinephrine-dopamine reuptake inhibitor (NDRI) and nicotinic antagonist, bupropion is a member of the class of aminoketones. Bupropion is used to treat major depressive disorder (MDD), is a medical aid for smoking cessation, and is also used as the alternative treatment of choice for MDD patients with sexual dysfunction caused by serotonin reuptake inhibitors (SSRIs) [1,2]. Some common unwanted effects include headache, dry mouth, nausea, dizziness, tremor, anxiety, insomnia, and constipation [2, 3]. Dystonia and other neurological toxicities, however, are rarely reported in the literature. Antipsychotics from the first generation tend to have a higher incidence of acute dystonia because of their higher potency [4]. Postural abnormalities and involuntary twisting movements brought on by chronic contraction of antagonistic muscle groups characterize acute dystonia. Depending on the drug, symptoms can appear anywhere from a few hours to a few days after dosing changes are made [4, 5].

## Case presentation

A non-diabetic 54-year-old woman with a family history of coronary artery disease (CAD) presented to the emergency department of a tertiary hospital. She reported a persistent contraction of her facial muscles, accompanied by temporomandibular joint (TMJ) pain and neck stiffness. She was immediately admitted and given an intravenous dose of 10 mg of diazepam. After reviewing her medical history, it was found that she was prescribed bupropion sustained release (SR) by her primary care physician for her chronic smoking. The patient was concerned about her risk of coronary artery disease and desired to adopt a healthy lifestyle and diet. She is not hypertensive and has a family history of coronary artery disease. She has been a smoker for the past three decades and currently consumes 22 cigarettes per day. She wishes to quit smoking and desires medical aid from her physician to assist her. She had no history of psychiatric illness and her score of zero on patient health questionnaire (PHQ-9) ruled out depression. She teaches at a school and is very happy with her life. Her primary care physician prescribed 150 mg of sustained release (SR) bupropion to help her quit smoking. She tolerated the dose well, so it was increased to 300 mg daily after three days, and she was instructed to report in one week.

However, one day after taking her first dose of bupropion 300 mg SR, she presented to our emergency department early in the morning with sustained muscle contraction of the left side of her face, resulting in facial dystonia involving the TMJ as well as left-sided neck stiffness. There were no head injuries or fever associated with the condition. Her lab parameters and serum electrolytes were unremarkable. She was immediately hospitalized and given 10 mg of intravenous diazepam. The patient had no history of such episodes and denied taking medications other than the recently prescribed bupropion. The medication was discontinued after a possible adverse reaction to bupropion was suspected. Her dystonia improved within a week after she stopped taking bupropion 300 mg SR daily. The patient was asked to undergo a re-challenge with medication, but she declined. She did not experience a recurrence of dystonia upon follow-up at two, four, and six weeks. Based on Naranjo's score of 6 and her sudden onset and remission of dystonia following bupropion intake and cessation, the primary diagnosis was dystonia induced by bupropion (Table 1).

**Table 1:** Naranjo Scale Of Adverse Reaction

Questions	Yes	No	Do not know	Patient
1. Are there previous conclusive reports on this reaction?	1	0	0	1
2. Did the adverse event appear after the suspected drug was administered?	2	-1	0	2
3. Did the adverse reaction improve when the drug was discontinued or a specific antagonist was administered?	1	0	0	1
4. Did the adverse event reappear when the drug was re‐administered?	2	-1	0	0
5. Are there alternative causes (other than the drug) that could on their own have caused the reaction?	-1	2	0	2
6. Did the reaction reappear when a placebo was given?	-1	1	0	0
7. Was the drug detected in blood (or other fluids) in concentrations known to be toxic?	1	0	0	0
8. Was the reaction more severe when the dose was increased or less severe when the dose was decreased?	1	0	0	0
9. Did the patient have a similar reaction to the same or similar drugs in any previous exposure?	1	0	0	0
10. Was the adverse event confirmed by any objective evidence?	1	0	0	0
Total score :				6

## Discussion

Dystonia is characterized by involuntary and persistent muscle contractions that manifest in a wide variety of abnormal twisting and repetitive movements and postures. Dopaminergic pathways in the brain are involved with the production of dystonia. Serotonin also interacts with these dopaminergic pathways [6]. A number of medications, such as antipsychotics and SSRIs, can have this effect [7].

Medication-induced focal dystonias are typically characterized by dramatic and twisting involuntary movements caused by head and neck muscle spasms and are occasionally accompanied by the clenching of the jaw, bruxism, and TMJ syndrome. Shortly after starting, increasing, or decreasing a medication's dosage can trigger acute dystonia [8]. Stimulation of serotonin on dopaminergic pathways in the central nervous system is the underlying cause of dystonia [6]. Acute dystonia typically manifests itself within the first 96 hours of starting treatment with antipsychotic medications or after a significant increase in the dose of such drugs [8]. Since bupropion is a dopamine and noradrenaline reuptake inhibitor, it is hypothesized that it may increase dopaminergic and noradrenergic activity, thereby causing dystonia [1, 2, 9]. We looked through the medical literature and found only a few isolated cases, such as one where acute dystonia symptoms developed after the patient stopped taking bupropion [8]. An increased dose of bupropion may also cause acute dystonia, according to some case studies [2, 6, 8-10].

In addition, Elyasi and Mahtiyan described a 34-year-old male patient with a painful neck spasm, which was sufficient to wake him up and induce dystonic distortion, followed by the administration of a single dose of up to 75 mg of bupropion [10]. Wang et al. also reported a patient with major depression and acute dystonia in two separate episodes after the patient suddenly stopped taking bupropion. The first episode occurred when the drug was suddenly switched from bupropion to duloxetine. In both instances, the symptoms, which included trismus, dysphagia, and torticollis, resolved after the reintroduction of bupropion or the injection of biperiden [11].

However, the possibility of Wilson's disease, hypocalcemia, tetanus encephalitis, catatonia, and conversion and malingering disorders should be considered in the differential diagnosis [8]. Acute dystonia is possible with concurrent use of bupropion and a drug that affects serotonin reuptake like an SSRI or buspirone. Buspirone and bupropion both affect the dopaminergic and serotonergic systems [9]. Anticholinergic medications like diphenhydramine, biperiden, and benztropine treat extrapyramidal symptoms (EPS) caused by drug side effects. Within 30 minutes, many patients report feeling better. Although some patients may require multiple administrations, long-term treatment is uncommon [12].

## Conclusions

Drug-induced focal dystonias have long been recognized as a side effect of certain medications in the past. Bupropion has rarely been identified as a causative factor of focal dystonias. Recognizing bupropion as a dystonia offender and discontinuing it immediately remains the most effective approach to treating culminating dystonias. The literature describes variable presentations of this dystonia alongside a wide range of dosages that are required to produce this effect. Henceforth, comprehensive studies aimed at exploring the subsequent clauses of bupropion need to be carried out.
